# Integration of robotic surgery into routine practice and impacts on communication, collaboration, and decision making: a realist process evaluation protocol

**DOI:** 10.1186/1748-5908-9-52

**Published:** 2014-05-02

**Authors:** Rebecca Randell, Joanne Greenhalgh, Jon Hindmarsh, Dawn Dowding, David Jayne, Alan Pearman, Peter Gardner, Julie Croft, Alwyn Kotze

**Affiliations:** 1 School of Healthcare, Baines Wing, University of Leeds, Leeds LS2 9JT, UK; 2 School of Sociology and Social Policy, University of Leeds, Leeds LS2 9UT, UK; 3 Department of Management, King’s College London, London SE1 9NH, UK; 4 Columbia University School of Nursing, 617 West 168th Street, New York, NY 10032, USA; 5 Center for Home Care Policy and Research, Visiting Nursing Service of New York, 5 Penn Plaza, New York, NY 10001, USA; 6 Leeds Institute of Biomedical and Clinical Sciences, University of Leeds, St. James’s University Hospital, Leeds LS9 7TF, UK; 7 Centre for Decision Research, University of Leeds, Leeds LS2 9JT, UK; 8 Institute of Psychological Sciences, University of Leeds, Leeds LS2 9JT, UK; 9 Clinical Trials Research Unit, University of Leeds, Leeds LS2 9JT, UK; 10 Department of Anaesthesia, Leeds Teaching Hospitals NHS Trust, Leeds General Infirmary, Leeds LS1 3EX, UK

**Keywords:** Process evaluation, Complex interventions, Realist evaluation, Robotic surgery

## Abstract

**Background:**

Robotic surgery offers many potential benefits for patients. While an increasing number of healthcare providers are purchasing surgical robots, there are reports that the technology is failing to be introduced into routine practice. Additionally, in robotic surgery, the surgeon is physically separated from the patient and the rest of the team, with the potential to negatively impact teamwork in the operating theatre. The aim of this study is to ascertain: how and under what circumstances robotic surgery is effectively introduced into routine practice; and how and under what circumstances robotic surgery impacts teamwork, communication and decision making, and subsequent patient outcomes.

**Methods and design:**

We will undertake a process evaluation alongside a randomised controlled trial comparing laparoscopic and robotic surgery for the curative treatment of rectal cancer. Realist evaluation provides an overall framework for the study. The study will be in three phases. In Phase I, grey literature will be reviewed to identify stakeholders’ theories concerning how robotic surgery becomes embedded into surgical practice and its impacts. These theories will be refined and added to through interviews conducted across English hospitals that are using robotic surgery for rectal cancer resection with staff at different levels of the organisation, along with a review of documentation associated with the introduction of robotic surgery. In Phase II, a multi-site case study will be conducted across four English hospitals to test and refine the candidate theories. Data will be collected using multiple methods: the structured observation tool OTAS (Observational Teamwork Assessment for Surgery); video recordings of operations; ethnographic observation; and interviews. In Phase III, interviews will be conducted at the four case sites with staff representing a range of surgical disciplines, to assess the extent to which the results of Phase II are generalisable and to refine the resulting theories to reflect the experience of a broader range of surgical disciplines. The study will provide (i) guidance to healthcare organisations on factors likely to facilitate successful implementation and integration of robotic surgery, and (ii) guidance on how to ensure effective communication and teamwork when undertaking robotic surgery.

## Background

The past two decades have seen a revolution in general surgical practice. In the 1990s, traditional open surgery was challenged by the introduction of laparoscopic techniques, initially for benign conditions, but later extended to the treatment of cancer. Instead of producing large abdominal wounds, the surgeon is able to perform operations using small ‘key-hole’ incisions, through which cameras and instruments are passed. This effectively removes much of the abdominal access trauma. The clinical benefits were soon realised, including less postoperative pain, shorter hospitalisation, quicker return to normal function, and improved cosmetic effect [1-3]. However, laparoscopic operations are technically more challenging than open surgery, as a result of the two-dimensional operative image, instrumentation with limited freedom of movement, and lack of tactile feedback. The uptake of laparoscopic surgery has therefore been slow; in 2003, the uptake in colorectal surgery was 5% and had increased to only 40% over the 9 years to 2011 [4], despite being recommended by the National Institute for Health and Care Excellence (NICE) since 2006. Robotic surgery offers to solve some of the limitations of the laparoscopic approach. A surgical cart carries four robotic arms, one of which holds the camera, while the other arms hold a variety of surgical instruments. These robotic arms are controlled by the surgeon remotely. The robot provides a stable camera image with three-dimensional field of view, with instruments that provide increased freedom of movement, and a digital platform that enables intuitive instrument handling, tremor elimination, and motion scaling. This enables the surgeon to achieve greater precision and control and simplifies many of the tasks that are difficult with traditional laparoscopy.

Enthusiasm for robotic surgery is expressed by both clinicians and policy makers [5-7]. Despite this, the many potential benefits robotic surgery offers for patients are currently not being realised to the full extent because of underuse of surgical robots [8]. Robotic surgery is a complex intervention, by which we mean that it is an intervention aimed at producing change in the delivery and organisation of healthcare services and which comprises a number of separate components that may act both independently and interdependently [9,10]. These components are not only technological but also organisational and social, and they can all impact the extent to which the technology is successfully introduced, as well as subsequent process and patient outcomes. The successful performance of a surgical operation is dependent on collaboration amongst staff from different professional groups, including surgeons, anaesthetists, nursing staff, and operating department practitioners (ODPs). There is a complex division of labour that requires the various team members to use their different skills collaboratively to accomplish a single, principal activity [11]. Reports of the use of robotic surgery suggest that a number of factors are important for successful integration, such as having a highly motivated [12] and/or dedicated robotic team [13-15] and additional staff [16]. Operating theatre (OT) staff consider teamwork skills to be critical for easing the integration of robotic surgery, as is having predefined protocols and explicit communication in the event of deviation from the protocol [17]. There is also acknowledgement that there is a learning curve for the whole team [18], not just the surgeon, and that the whole team requires training [19]. However, such recommendations come from small case series (descriptive non-randomised studies) undertaken in single institutions, typically by dedicated robotic surgery enthusiasts [3], so that little is known about the contextual factors that are necessary for the successful integration of robotic surgery into healthcare organisations more broadly.

Existing evaluations of robotic surgery also fail to consider the impact of robotic surgery on communication, teamwork, and decision making in the OT. Robotic surgery significantly changes the spatial configuration in the OT, with the surgeon at a distance from the patient and team. The surgeon’s visual attention is focused on the three-dimensional image provided by the robot, inhibiting face-to-face communication during the operative part of the procedure. More generally, the size of the robot introduces physical space constraints, resulting in a new choreography of movement around the patient [17]. The impact of this change in spatial configuration on communication and teamwork in the OT is not a topic that has been explored in evaluations of robotic surgery, which typically focus on the role of the surgeon [20]. The spatial configuration of team members and technology in the OT influence the gathering of information that is used to inform decision making [21,22]. More generally, the spatial configuration of OT teams is not arbitrary but affords particular views of the patient, the rest of the team, and different tools and technologies, with the result that different team members have access to different information to inform their decision making [23]. The nature of the decision making tasks of the OT team may be impacted with robotic surgery. Surgeons report a sense of both physical and psychological isolation from the patient in robotic surgery, and he/she is more dependent on the rest of the team communicating the status of the patient to maintain situation awareness [17,24]. Consequently, it has been argued that decision making in robotic surgery is essentially collaborative [24]. In the event of a complication, more of the burden falls on the rest of the team to respond, increasing the importance of the team having a shared situation awareness of what is happening in the operative field and how far they are through the procedure [17].

Two small studies have looked specifically at differences in communication between laparoscopic and robotic surgery [25-27]. Both studies found a significant increase in oral communication between the surgeon and the rest of the team in robotic surgery, particularly in relation to the orientation and localisation of organs and the manipulation of instruments, with the effect found to be more pronounced in teams that have less experience in robotic surgery [25]. If use of robotic surgery interferes with standard practices of coordination among the OT team, the achievement of seamless, efficient and timely teamwork may be hampered. It is important to understand any change in communication patterns because of the well-documented relationship between communication and patient safety, with failures in communication and teamwork being identified as key factors in adverse events in the OT [28]. Communication and teamwork around robotic surgery are likely to be influenced by processes associated with the introduction of robotic surgery, such as training and changes in team structure, but equally the integration of robotic surgery in surgical practice may be dependent on the extent to which it is consistent with existing practices for coordination.

For robotic surgery to provide the most benefit for patients, it is first necessary to understand the organisational and social factors that support the successful integration of robotic surgery, by which we mean that it becomes embedded into surgical practice, being used routinely and successfully for surgical operations where it offers advantages to the patient. It is also necessary to understand the impacts of robotic surgery on communication, teamwork, and decision making in the OT and how OT teams manage those impacts.

### Aims and objectives

The aim of this project is to understand how and in what circumstances robotic surgery produces both intended and unintended outcomes. This will be achieved through a realist process evaluation, running alongside an existing randomised controlled trial (RCT) comparing laparoscopic and robotic rectal cancer surgery for the curative treatment of rectal cancer. The study has the following research objectives:

1. To contribute to the interpretation and reporting of the trial results by investigating how variations in implementation of robotic surgery, and the context in which robotic surgery is implemented, impact on outcomes such as operation duration, conversion to open surgery, and complications;

2. To produce actionable guidance for healthcare organisations on factors likely to facilitate successful implementation and integration of robotic surgery; and

3. To produce actionable guidance for OT teams on how to ensure effective communication and teamwork when undertaking robotic surgery.

## Methods and design

### Overall design

We will undertake a realist process evaluation that will run alongside ROLARR (RObotic versus LAparoscopic Resection for Rectal Cancer), an international, multicentre prospective, randomised, controlled, unblended parallel-group trial, where the primary outcome is conversion to open surgery (as an indicator of technical difficulty) [29]. Process evaluations are predominantly qualitative studies that are typically undertaken alongside a trial, but may be undertaken in preparation for a trial or after a trial [30], and explore how the intervention is implemented [31]. This involves defining the active components of the intervention and investigating contextual factors that affect its implementation.

Realist evaluation is increasingly popular as a method for evaluating the implementation of complex interventions in healthcare [32-36] and has been applied in a variety of fields within health systems research [37]. It does not employ particular methods of data collection but offers a framework for understanding for whom and in what circumstances complex interventions work. It involves building, testing and refining the underlying assumptions or theories of how the intervention is supposed to work [38]. Realist evaluation can complement RCTs in a number of ways [39]. Previously, it has been used in preparation for an RCT, to develop hypotheses that were then tested in the trial [40,41], and for a process evaluation following an RCT, to understand the results of the trial, in terms of how and why the outcomes were achieved [42,43].

### Phase I. Formulation of CMO configurations

The unit of analysis in realist evaluation is not the intervention but the theories concerning the mechanisms through which the intervention produces certain outcomes in particular contexts. A first task is to identify these theories. In Phase I, the first ‘theory elicitation’ stage of a realist synthesis will be undertaken to catalogue stakeholders’ theories concerning how robotic surgery becomes embedded into surgical practice and its impacts. Such theories are to be found in guidance documentation (*e.g.*, for robotic surgery), position papers, professional journals such as the ‘Health Service Journal’ and the ‘Nursing Times,’ publications of the Royal Colleges, blogs, thought pieces, advocacy pieces, and critical pieces, and so the review will focus on this grey literature. The output of the review will be a series of candidate theories in the form of Context-Mechanism-Outcome (CMO) configurations.

Telephone interviews will be conducted with staff in National Health Service (NHS) hospital Trusts (providers) in England that are using robotic surgery for rectal cancer resection. This will include Trusts that are participating in the trial and those that are not. Interviews will be semi-structured and conducted using the ‘teacher learner cycle’ [44]. Here, the interviewer describes, through their interview questions, the candidate theories to the interviewee, who is then invited to comment, expand and discuss the theories based on their experience of the intervention. Through this process, the interviewer channels the interviewee’s responses to the task of developing and refining the theories. The interviewer proceeds to formalise the interviewee’s theories, based on the information they have given, and the interviewee is then invited to comment on that formalisation. Consequently, the interview is a vehicle for enabling key participants to revise and expand the theory and generate new theories. It is essential that the study captures the perspectives of all professional groups that may influence the effectiveness of the introduction of robotic surgery [24]. Interviews will first be held with one of the surgeons in each Trust and, through them, we will identify other members of the OT team to interview (surgeons, anaesthetists, theatre nurses, ODPs, trainee surgeons) as well as hospital administrators and managers who were involved in the introduction of robotic surgery into the Trust. All interviews will be audio recorded and transcribed verbatim.

An iterative approach to data collection and analysis will be taken in this phase, to support the gathering of further data on emergent themes. Interview transcripts will be entered into a qualitative software programme (NVivo 10) for indexing. Thematic analysis will be used to analyse the data [45]. Following the realist strategy, indexing of the data will focus on identifying interviewees’ accounts of how outcome patterns are formed by mechanisms and contexts [43]. In addition, codes developed inductively will be used to index the data. Similarities and differences in the stakeholder theories will be identified and used to further refine the emerging CMO configurations. Key CMO configurations will then be selected for empirical testing in Phase II.

### Phase II. Empirical testing of CMO configurations

Four case sites will be selected, three of which will be participants in the ROLARR trial and one which is not (on the basis that only looking at sites in the trial would limit the generalisability of the findings, as sites in the trial could be considered to have successfully embedded robotic surgery into their practice). Case sites will be purposively sampled to ensure variation in the contextual factors identified as being significant in Phase I of the research. These are likely to include: process through which the technology was introduced (organisation led vs. clinician led); level of experience with robotic surgery; and whether dedicated robotic teams are employed.

In realist evaluation, a mixture of qualitative and quantitative methods is important to gather data on the mechanisms and contexts of an intervention as well as its impacts [46]. Data will be collected using a range of methods, which are flexible enough to allow exploration of a variety of CMO configurations. However, the data collection protocol will be revised and further specified at the end of Phase I in light of the theories to be tested.

### Data collection

#### Structured observation

Teamwork during operations will be assessed using OTAS (Observational Teamwork Assessment for Surgery) [47]. OTAS comprises ratings on five team behaviour constructs: (i) Communication; (ii) Coordination; (iii) Cooperation and back up behaviour; (iv) Leadership; and (v) Team monitoring and situation awareness. These behaviours are assessed during observation of the surgery, with each behaviour scored on a 7-point scale. OTAS distinguishes between different subteams in the OT (surgeons, anaesthetists, and nurses) and different phases of a procedure (pre-, intra-, and post-operative). For one operation, a total of 45 behavioural ratings are generated (5 behaviour constructs × 3 subteams × 3 phases). Researchers will also record additional information for each operation, such as the team composition, each team member’s level of experience of robotic surgery, duration of each phase of the operation, and whether there was a conversion to open surgery.

#### Video

To facilitate a close consideration of robotic surgery in use, all operations that are observed will be video recorded. Video recording has been highlighted as an important tool for understanding safety in the OT [48] and has been used successfully in a number of studies concerned with the impact of communication and teamwork on surgical performance [49-51]. The use of two video cameras will allow for the capture of the surgeon’s perspective on the surgical scene and the wider conduct of OT team members.

#### Ethnographic observation

Ethnography has been argued as an essential approach for studying the introduction of technology into healthcare settings [52]. It is an important complement to the structured observations, allowing unanticipated yet significant behaviours and interactions that fall outside the scope of the observation tool to be recorded in the researcher’s field notes. In addition, ethnographic observation is important for getting a sense of how what happens in the OT fits within the broader context of work and for capturing those aspects of the context that cannot easily be measured, such as the culture of an organisation. Following in the ethnographic tradition, the researchers will, at least in the early stages of the study, keep the scope of the notes wide on the basis that what previously seemed insignificant may come to take on new meaning in light of subsequent events [53] and should give special attention to the indigenous meanings and concerns of the people studied [54]. In addition, the researchers will record incidents of observer effects (*e.g.*, participants asking ‘What are you writing?’) to allow analysis of whether participants’ awareness of the researchers’ presence changed over time [55]. Field notes will be written up as soon after data collection as possible.

### Interviews

Interviews will be semi-structured and will seek to gather data on those outcomes that cannot easily be gathered by other means, particularly those relating to the perceptions of members of the OT team (*e.g.*, level of enthusiasm for robotic surgery, perceptions of teamwork, and perceptions of robotic surgery as an opportunity for training). In addition, due to the infrequency of such events, we are unlikely to see many conversions to open surgery or complications (conversion to open surgery is expected to occur in less than 5% of operations), so we will gather OT team members’ accounts of the reasons for such conversions. All interviews will be audio recorded and transcribed verbatim.

### Data analysis

As in Phase I, an iterative approach to data collection and analysis will be taken. The overall approach to analysis will involve initial comparisons in the processes and outcomes of interest (*i.e.*, those specified in the CMO configurations) between laparoscopic and robotic surgery, before using the data from the robotic surgery operations to test the CMO configurations. We will try to draw on multiple sources of data to test each configuration. Thus, it is through testing the CMO configurations that the various sources of data will be integrated. Table 1 provides some example theories, based on our current understanding of the literature, and indicates what data could be used for testing each theory.

**Table 1:** Example theories presented as CMO configurations, with data to support testing

**Context**	**+**	**Mechanism**	**=**	**Outcome pattern**	**Data**
Experienced teams	+	Knowledge of how to overcome difficulties in set-up and positioning of the robot	=	Easier access to patient; Reduced operation duration; Reduced conversion to open surgery/complications	Video recordings, operation duration, interviews
Whole OT team involved in implementation	+	OT team perceive benefits for patients	=	Increased motivation among team members to work together to develop solutions to problems	Video recordings, OTAS, interviews
Physical separation between surgeon and OT team	+	OT team less aware of surgeon’s actions	=	Reduced coordination; Increased operation duration	OTAS, video recordings, operation duration
Dedicated robotic teams	+	Develop strategies to deal with physical separation	=	Effective coordination, teamwork, communication; Reduced operation duration	OTAS, video recordings, operation duration
Surgical trainee as part of team	+	Physical separation and different views of operative field makes it harder for surgeon to explain what is happening and monitor trainee’s understanding	=	Reduced satisfaction amongst surgeon and trainee in robotic surgery as opportunity for training	Video recordings, interviews
More experienced teams	+	Understand need to support surgeon’s situation awareness	=	Increased verbal communication of patient state to surgeon; Increased situation awareness of surgeon	Video recordings, OTAS

### OTAS

Initial analysis of OTAS scores will use a mixed model analysis of variance (ANOVA), with case site and sub-team as between-subjects factors and surgery type (laparoscopic vs. robotic), phase, and behaviour as within-subjects factors [47]. We will then test the CMO configurations. For example, this could involve checking for a difference in the overall OTAS score in those case sites where the OT team had been involved in implementation compared to those case sites where they were not involved.

### Video

We will follow standard methods outlined in the literature on video and the analysis of work practice [56], which draw heavily on ethnomethodology [57] and conversation analysis [58]. This analysis will be undertaken by multiple members of the research team, and team ‘video review sessions’ will be a routine and regular feature of project work. Given the complex and highly specialised character of the setting, specific video extracts and preliminary analytic observations will be discussed with participants at each case site (and the clinical members of the research team) to ensure that the findings are robust, to generate alternative avenues for inquiry and to discuss implications of the findings for practice.

### Ethnographic field notes and interview transcripts

Field notes and interview transcripts will be entered into NVivo 10 for indexing and will be analysed using the methods outlined for the analysis of the interview data in Phase I. This will identify data to support or refute particular CMO configurations, as well as identifying additional CMO configurations.

### Phase III. Assessing the generalizability of CMO configurations

Interviews will be conducted at the four case sites with participants from different surgical disciplines to assess the generalisability of the CMO configurations that result from Phase II and to further refine them to reflect the experience of a broader range of surgical disciplines. The interviews will be conducted using the ‘teacher learner cycle’, as in Phase I. Again, a range of participants (surgeons, anaesthetists, theatre nurses, ODPs, trainee surgeons) from a range of surgical disciplines in which robotic surgery is being used will be included. All interviews will be audio recorded and transcribed verbatim. Interview transcripts will be analysed using the methods outlined for the analysis of the interview data in Phase I.

### Ethical considerations

Phase I of the study has been approved by the University of Leeds School of Healthcare Research Ethics Committee (SHREC/RP/339). NHS Research Ethics Committee approval for Phases II and III of the study has been granted (13/YH/0153).

## Discussion

This paper describes the protocol for a realist process evaluation that uses multiple methods to understand how and under what circumstances robotic surgery is effectively introduced into routine practice; and how and under what circumstances robotic surgery impacts teamwork, communication and decision making, and subsequent patient outcomes. The study will provide actionable guidance for healthcare organisations on factors likely to facilitate successful integration of robotic surgery and for OT teams on how to ensure effective communication and teamwork when undertaking robotic surgery.

There has been much work in recent years on methods for evaluating complex interventions [9,10,31,59,60]. At the same time, there has been work on methods for assessing surgical innovations, on the basis of their inherently complex nature [61,62], with some arguing for the inclusion of qualitative data collection alongside RCTs of surgical procedures [63]. Process evaluations are recommended when evaluating complex interventions because, although the RCT design remains as the most reliable method of determining effectiveness [9], it is necessary to understand the mechanisms through which the intervention achieves its outcomes [59]. Without this, effective aspects of the intervention may go unmeasured, raising concerns about the validity and reliability of the results of an evaluation [64] and preventing replication [65]. Process evaluations are particularly important in multicentre trials in which the intervention may be implemented in different ways [31]. Understanding how the components of the intervention and the context vary across sites can assist in interpreting differences in results.

However, the ability of process evaluations to shed light on how an intervention achieves its outcomes has been constrained by a tendency to focus on intervention components rather than on mechanisms through which the outcomes of interest are generated [66]. Where mechanisms are examined, the tendency has been to develop hypotheses about the relationship between intervention components, context, and outcomes, without explicitly testing those hypotheses. The advantage of realist evaluations is that they explain how different contexts trigger particular mechanisms which, in turn, give rise to certain outcomes. Thus it increases the specificity of our understanding of the relationship between context, mechanisms and outcomes.

Discussions with the ROLARR team suggest that the process evaluation will be able to provide insight to support the interpretation of the trial data in terms of understanding any increase in operation duration in robotic surgery and what leads to conversion to open surgery or to complications. In developing the CMO configurations and selecting CMO configurations for testing, attention will be paid to the contexts and mechanisms that may relate to such outcomes. Subject to recruitment, from the data collected in Phase I, we will be able to provide an account of the different ways in which robotic surgery was implemented in all English ROLARR sites, in terms of components of the intervention. Combining these data with the outcome data from the trial will allow for testing of CMO configurations that relate to the trial outcomes and can assist in understanding differences between sites. Inclusion of these findings into the reporting of the ROLARR trial will provide important information for healthcare organisations that are considering introducing robotic surgery.

Despite the successful use of realist methods in process evaluations alongside cluster RCTs of complex interventions and claims by authors of such studies that one of the strengths of realist evaluation is its ability to deal with complexity [37], the role that realist approaches can take in understanding the outcomes of RCTs of complex interventions has been disputed [66-68]. Some would say that realist evaluation is epistemologically antagonistic to the use of RCTs [69], and it has also been claimed that realist evaluation does not deal well with complex multi-site interventions with multiple outcomes [69]. Certainly, we would agree with those who have called for further scholarly debate on the place of realist evaluation in RCTs [68], and the proposed study will contribute to that debate, as well as contributing to the development of methods for the evaluation of surgical innovations.

## Abbreviations

CMO: Context-Mechanism-Outcome; NHS: National Health Service; OT: Operating theatre; OTAS: Observational Teamwork Assessment for Surgery; RCT: Randomised controlled trial; ROLARR: RObotic versus LAparoscopic Resection for Rectal Cancer.

## Competing interests

The authors declare that they have no competing interests.

## Authors’ contributions

RR is the principal investigator for the study. She conceived, designed, and secured funding for the study in collaboration with JG, JH, DD, DJ, AP, PG, JC and AK. All authors provided input into various aspects of the study design and revised drafts of the protocol. RR led the writing of this protocol manuscript. All authors read and approved the final manuscript.
